# Revealing Subtle Functional Subgroups in Class A Scavenger Receptors by Pattern Discovery and Disentanglement of Aligned Pattern Clusters

**DOI:** 10.3390/proteomes6010010

**Published:** 2018-02-08

**Authors:** Pei-Yuan Zhou, En-Shiun Annie Lee, Antonio Sze-To, Andrew K. C. Wong

**Affiliations:** 1 VaryWave Technology Co., Ltd., 538A, Core Building 2, Hong Kong Science Park, Shatin, NT, Hong Kong; choupeiyuan@gmail.com; 2 VerticalScope Inc., 111 Peter Street, Suite 900, Toronto, ON M5V 2H1, Canada; alee@verticalscope.com; 3 Systems Design Engineering, 5th, 6th Floor, 200 University Avenue West, University of Waterloo, Waterloo, ON N2L 3G1, Canada; hy2szeto@uwaterloo.ca

**Keywords:** residue association, pattern discovery, disentanglement, protein, class A scavenger receptors

## Abstract

A protein family has similar and diverse functions locally conserved as aligned sequence segments. Further discovering their association patterns could reveal subtle family subgroup characteristics. Since *aligned residues associations* (ARAs) in Aligned Pattern Clusters (APCs) are complex and intertwined due to entangled function, factors, and variance in the source environment, we have recently developed a novel method: Aligned Residue Association Discovery and Disentanglement (ARADD) to solve this problem. ARADD first obtains from an APC an ARA Frequency Matrix and converts it to an adjusted *statistical residual vector* *space* (SRV). It then disentangles the SRV into Principal Components (PCs) and Re-projects their vectors to a SRV to reveal succinct orthogonal AR groups. In this study, we applied ARADD to class A scavenger receptors (SR-A), a subclass of a diverse protein family binding to modified lipoproteins with diverse biological functionalities not explicitly known. Our experimental results demonstrated that ARADD can unveil subtle subgroups in sequence segments with diverse functionality and highly variable sequence lengths. We also demonstrated that the ARAs captured in a Position Weight Matrix or an APC were entangled in biological function and domain location but disentangled by ARADD to reveal different subclasses without knowing their actual occurrence positions.

## 1. Introduction

Proteins from the same family have similar but also diverse functions [1]. Hence, discovering conserved yet varied sequence patterns with various subgroup characteristics is important for understanding the protein functionality of a protein family. However, existing broad and imprecise grouping definition and processes neglect the diversity of certain protein families [1]. One protein family may contain members that are strikingly variable in sequence length, three-dimensional structure, and hence biological function, where similar and diverse functional domains may reside in different sequence locations within the family. For example, class A scavenger receptors (SR-A) [1], biologically important for binding on modified lipoproteins, are complex particles composed of multiple proteins that transport all fat molecules (lipids) around the body within the water outside cells to promote macrophage differentiation into foam cells, leading to chronic conditions such as atherosclerosis [2]. SR-A is a diverse family of proteins classified based on their ability to bind modified lipoproteins [3]. Although the five members (Marco, Sra, Scara3, Scara4, Scara5) [1] of this family could bind modified lipoproteins, they are different in terms of their sequence patterns, locations, structures, and hence functions. For instance, within the same family, their protein length varies from 451 to 732 residues with the functional domains residing in different sequence locations. Thus SR-A is a protein family with conserved yet diverse function subgroups, ideal for exploration.

In order to reveal the functional subgroup characteristics of conserved sequence patterns corresponding to the diverse members of a protein family, we need mathematical transformations to disentangle the intriguing functionality related to conserved functional regions to reveal subgroups not explicitly manifested from the data. To this end, we have developed a novel method, known as Aligned Residue Association Discovery and Disentanglement (ARADD) [4], based on our previous work Attribute Value Association Discovery and Disentanglement (AVADD) [5], to discover and then disentangle the statistical representation of the Aligned Residue Associations (ARAs) derived from Aligned Pattern Clusters (APCs) for revealing their subgroups and subgroup characteristics.

In this study, we conducted experiments on SR-A, and found that the sequence patterns that are aligned and clustered into an APC via sequence similarity could be entangled in biological functions and domain location. Hence, we applied ARADD [4] to disentangle the statistics so as to reveal the ARs and ARA Clusters (Patterns) corresponding to their functionality and residing location in the family. We further showed that even the ARAs captured in a Position Weight Matrix (PWM) [6] discovered by the famous motif discovery MEME [7] could be entangled in biological functions and domain location. We then demonstrated the effectiveness of ARADD [4] in disentangling ARA patterns to reveal functional subgroups. The major contribution of our study is three-fold.

1. We were able to show that sequence patterns in the representation model, could be mixed or entangled in functionality and location through the study of the SR-A data.
2. We validated that ARADD [4] could reveal functional subgroups and subgroup characteristics of APCs and locate their residing domains through the case study on SR-A. Understanding its subgroup characteristics could also render new knowledge for gene therapy applications [2].
3. We demonstrated that the sequence patterns captured by the Position Weight Matrix (PWM) [6] could be entangled in biological functions and domain location, and they needed to be disentangled by an effective method such as ARADD [4].

The focus of this paper, apart from introducing ARADD [4] and emphasizing its novelty, is to produce a succinct analysis of subgroup characteristics obtained through pattern disentanglement, via the case study on SR-A. To our knowledge, no studies have reported similar experimental results.

The paper layout is as follows. Section 2 does a literature survey. Section 3 explains the methodology. Section 4 describes the materials and Section 5 illustrates the experimental results. Section 6 outlines the biological significance. Section 7 summarizes and concludes the research.

## 2. Related Work

Traditionally, computational sequence analysis methods have been developed to identify conserved sequence patterns from a protein family. Methods such as Multiple Sequence Alignment (MSA) [8] are only suitable for globally homologous sequences with a high level of sequence similarity [9], and motif discovery [10]; another method, is based on probabilistic model (such as position weight matrix [6]) which assumes independence between residue columns to represent the conserved sequence patterns. Such independence assumption is unrealistic in many cases, where correlation of residues along the sequence is commonly observed [11,12].

Pattern discovery is an essential element in predictive analytics [13,14] for knowledge discovery and analysis. Its essence is to discover patterns (motifs) occurring in the data to reveal association patterns for interpretation and classification [15]. Hence, we develop an algorithm to obtain APCs [16,17] which capture functional residue association and site conservation. Since APCs contain aligned residues in strong statistical association sequence patterns, this representation is more knowledge-rich [16,17] when compared with MSA and probabilistic models. Hence, APCs reveal locally conserved yet diverse function patterns of protein families. APCs can reveal biological function in conserved regions of protein families.

Association rule mining [18] is the most well-known methodology for mining item sets in relational dataset in the area of data mining. Algorithms such as Apriori [19] and FP-growth [20] can be applied for capturing associations from relational dataset. However, frequent patterns discovered by the above algorithms are extremely sensitive to threshold settings. Our new method, Aligned Residue Association Discovery and Disentanglement (ARADD), evolved from our AVADD [5] method, and is proposed to solve this problem. ARADD is able to reveal residue association patterns in different orthogonal PCs and *Re-projected SRVs* (RSRVs). ARADD is able to correlate different functionalities based only on the confidence intervals. As demonstrated in the results reported in this paper, ARADD achieves stable and succinct results in a simple fashion.

As observed in our recent paper [5], a challenging problem encountered when discovering association patterns is that the association could be masked or obscured in the data due to the entanglement of unknown factors in their source environment. To resolve this problem for general relational datasets, we developed a novel method known as AVADD in our previous work [5]. In this paper, we transformed the existing methodology to discover and disentangle ARAs from APCs. The reasons are as follows: (1) the aligned columns (sites) in an APC can be treated as attributes of relational dataset; (2) the residing residues on these sites can be treated as attribute values; (3) the residue associations in an APC can be treated as attribute value associations. The extended ARADD from AVADD [5] could discover and disentangle ARAs from APCs as if AVADD [5] could do that on *attribute value associations* (AVAs) from a relational dataset. This is the most game-changing part of ARADD in comparison with existing methods. Due to such capability, subtle entangled subgroup characteristics masked or conspicuous in APCs can be revealed. To the best of our knowledge, only ARADD could disentangle such ARA patterns in APCs while no other reported methods could.

In summary, compared to the above-mentioned algorithms, ARADD solved the most difficult problems in discovering and analyzing subgroup characteristics of APCs containing entangled associations and the variation among them in their aligned sequence patterns. We should conclude that: (1) local associations may occur in different sequence locations or functional domains; (2) subgroups with similar patterns (motifs) could have small differences in functionality; (3) similar functionality may occur in different function groups and domains; and (4) multiple functionalities may occur within a functional group dominated by a key function. We refer to such entwined phenomena as the results of entangled ARA patterns.

## 3. Methods

The method used in this paper is evolved from AVADD [5], which was developed by our team. It was used for discovering and disentangling AVAs from mixed-mode relational datasets (RDS) [5] very successfully. In this study, we extended AVADD to discover and disentangle the statistical representation SRV of the ARAs obtained from APCs to reveal their subgroups and subgroup characteristics as well as to locate their functional domains.

**Figure 1:**
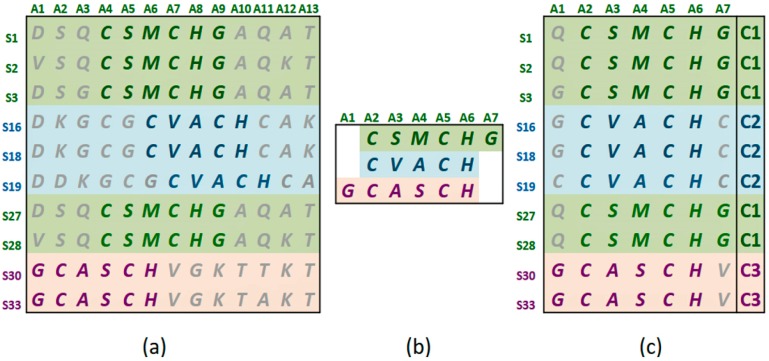
Protein Sequence and APC Data (**a**) Protein sequence dataset with high order association patterns (in bold) discovered by [11,12] with top row = aligned sites; fist column = sequence ID; (**b**) Aligned Pattern Cluster Pattern Space (*APC-P*) obtained using [16,17]; (**c**) APC Data Space (*APC-D*). The last column is the class labels.

By Aligned Pattern Cluster (APC) [16,17], we mean an array of sequence segments containing a cluster of aligned statistically significant sequence patterns grouped together according to their similarity though alignment [16,17]. Figure 1a shows the statistically significant sequence patterns discovered from the sequence data. Figure 1b represents the pattern space of the APC when the discovered patterns are aligned. Figure 1c is referred to as the data space of the APC showing all the sequence data segments containing the patterns in the APC with their pattern sequence ID and head position registered. Through the data space of APCs, any ARs or ARAs of an APC identified in any PCs and RSRVs, respectively, can be located.

**Figure 2:**
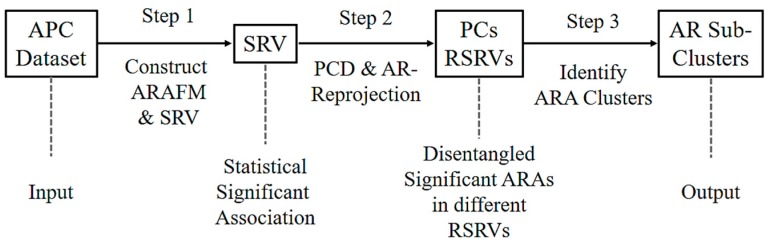
An overview of the proposed algorithm of Aligned Residue Association Discovery and Disentanglement (ARADD).

Figure 2 shows the three major steps of ARADD. (1) ARADD converts an APC into an ARA Frequency Matrix (ARAFM) (Definition 1). It then converts the ARAFM into an Adjusted Statistical Residual Space (SRV) so that each frequency entry in ARAFM is transformed into a Statistical Residual (SR) that accounts for the deviation of the frequency of occurrences of the ARA from the expected frequenting if the associations were random. In the SRV, each row represents a vector of an AR (referred to as an AR-vector or a-vector) whose coordinates represent the SRs of that AR associated with other ARs corresponding to the column AR-vectors; (2) Next, ARADD conducts the Principal Component Decomposition on the SRV to obtain the top Principal Components (PCs) ranked according to their variance, and then projects all the AR-vectors in the SRV onto the PC axis. The new SRV containing the vector projections on the PC is referred to as the Re-projected SRV. The new set of coordinates of these projections reflects the SRs of that AR associating with other ARs corresponding to the column vectors; (3) Finally, for each PC, ARADD identifies the distinct ARs and/or AR clusters with variance ≥1.0 from the center and then obtains the SR value of the ARA between the ARs within each AR clusters in the RSRV to reveal the association patterns. 

**Figure 3:**
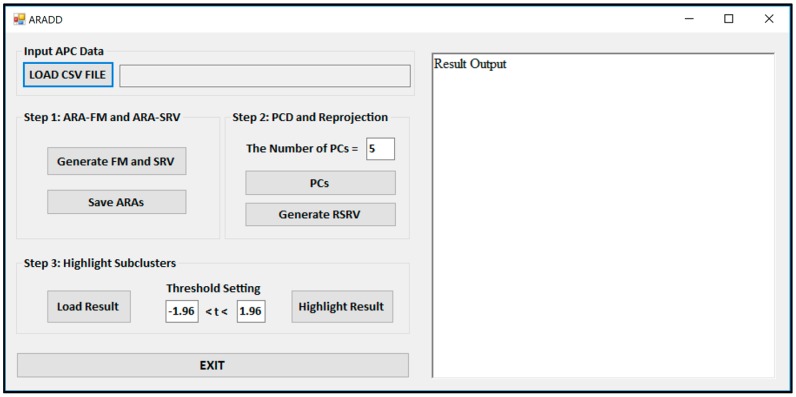
An example of the intermediate process and data produced by our Attribute Value Association Discovery and Disentanglement (AVADD) prototype via an Interactive Decision Support GUI. In the main ARADD GUI, the left-hand side shows the process of AVADD and the right-hand side shows the output result of each step but not displayed in this figure.

Figure 3 shows the Graphical User Interface (GUI) of the ARADD software. After loading the CSV file of an APC; pushing the button labeled “Generate FM and SRV” will create both the ARAFM and SRV for the APC. The process in Step 2 will generate the set of top PCs and their corresponding RSRVs according to the number of PCs or percentage of the variance assigned in the box. The process in Step 3 will highlight the sub-cluster results according to the assigned confidence interval in the box.

We can represent an APC dataset by M amino acids (residues) on N amino acid sites, denoted as *A* = {$A_{1},\ldots A_{n}\ldots A_{N}$}. Each amino acid site $A_{n}$ can assume a categorical value as a residue type. Thus $A_{n}$ contains $I_{n}$ values, denoted as $A_{n}=\left\{A_{n}^{1},A_{n}^{2},\ldots A_{n}^{I_{n}}\right\}$. Hence, *I* = $\overset{N}{\underset{n=1}{\sum }}I_{n}$ represents the total number of residue types (values) of all sites in an APC.

ARA represents the residue associations between residues on different aligned columns of the APC (i.e., the residue pairs). The ARA, $\left(A_{n}^{i}↔A_{n^{\prime }}^{j}\right)$ represent the residue association between two aligned residues with the aligned site positions *n* and *n*’ and the residue types *i*, *j* respectively.

**Definition** **1.** ARA Frequency Matrix. An ARAFM is a matrix of frequency counts of ARA between two aligned amino acids (residues) on two aligned sites, say $\left(A_{n}^{i}↔A_{n^{\prime }}^{j}\right)$, within the same protein sequence. We denote the frequency by $FM\;\left(A_{n}^{i}↔A_{n^{\prime }}^{j}\right)$, where $A_{n}^{i}$ represents the residue of i^th^ type on the n^th^ aligned site in the APC, and $A_{n^{\prime }}^{j}$ represents the residue of the j^th^ type on the n′^th^ aligned site (n $\neq n^{\prime }$). Hence, ARAFM is an I × I matrix.

Now we shall describe the operations associated with each step in the ARADD GUI.

Step 1: Construct Adjusted Statistical Residual Vectors Space (SRV):

The button “Generate FM and SRV” in Figure 3 is used to construct the ARAFM first and then to convert the ARAFM into SRV (Definition 2).

**Definition** **2.** ARA Adjusted Statistical Residual Vector Space. An SRV is a vector space such that the j^th^ jjas coordinate of its i^th^ iithat row vector (a-vector or more precisely $A_{n}^{i}-$vector), corresponding to the i^th^ type residue on the n^th^ site in the APC. We denote the n’ coordinate of the $A_{n}^{i}-$vector by $SRV_{A_{n’}^{j}}$ which is the SR obtained from $FM\left(A_{n}^{i}↔A_{n^{\prime }}^{j}\right).$ Hence, an SRV can be expressed as a set of row vectors: SRV = < $SRV_{A_{1}^{1}},\ldots SRV_{A_{1}^{I_{1}}},\ldots SRV_{A_{n}^{I_{n}}}\ldots ,SRV_{A_{N}^{I_{N}}}$ >, where I = $\overset{N}{\underset{n=1}{\sum }}I_{n}$ is the total number of ARAs, and $I_{1}$ is the total number of unique SRs for the first aligned column ($A_{1}$), and $I_{n}$ is the number of unique amino acid for the n^th^ aligned column ($A_{n}$). An a-vector is hence denoted as $SRV_{A_{n}^{i}}$ = $\{SR(A_{n}^{i}↔A_{1}^{1}$),… $SR(A_{n}^{i}↔A_{1}^{I_{1}}$),… $SR(A_{n}^{i}↔A_{N}^{I_{N}}$)}, where $SR(A_{n}^{i}↔A_{n^{\prime }}^{j}$) represents the statistical residual for ARA ($A_{n}^{i}↔A_{n^{\prime }}^{j}$), and $SR(A_{n}^{i}↔A_{n}^{i}$) = 0.

By replacing each ARA frequency with its *Adjusted Standard Statistical Residual* (SR), we can construct the SRV. We treat SRV as a vector space such that each row is taken as a row vector (AR-vector or a-vector) with its coordinates representing the SR of its ARAs associating with other ARs denoted by the column a-vectors.

To obtain statistically significant information from an APC, we transform an ARAFM into a SRV by converting each ARA frequency $FM\left(A_{n}^{i}↔A_{n^{\prime }}^{j}\right)$ into an SR, denoted as $sr(A_{n}^{i}↔A_{n^{\prime }}^{j}$).

(1) $$sr\left(A_{n}^{i}↔A_{n^{\prime }}^{j}\right)=sr_{ij}=\frac{o_{ij}-e_{ij}}{\sqrt{e_{ij}}}$$

where $o_{ij}$ represents the total number of occurrences when $A_{n^{\prime }}=A_{n^{\prime }}^{j}$ and $A_{n}=A_{n}^{i}$; $e_{ij}$ = $\frac{\sum_{u=1}^{J}o_{iu}\sum_{u=1}^{I}o_{uj}}{M}$, where $\overset{J}{\underset{u=1}{\sum }}o_{iu}$ represents the total number of counts when $A_{n^{\prime }}=A_{n^{\prime }}^{j}\overset{I}{\underset{u=1}{\sum }}o_{uj}$ represents the total number of counts when $A_{n}=A_{n}^{i}$ and *M* is the total number of records.

To reveal the statistical significance of an ARA, here, $sr_{ij}$ measures the deviation of the observed frequency, $o_{ij}$, of the ARA from its default model $e_{ij}$ assuming that the occurrence is a random association. Unlike the standardized residual, the Adjusted Statistical Residual considers the overall size of the sample and gives a more accurate indication of how far the observed count is from the expected count. So, in this paper we abbreviate Adjusted Statistical Residue as “*SR*”, to be consistent with our previous paper [5]. We denote *SR* as $SR(A_{n}^{i}↔A_{n^{\prime }}^{j}$), which is the significance of ARAs. The value of $SR\left(A_{n}^{i}↔A_{n^{\prime }}^{j}\right)$ is calculated using Equation (2).

(2) $$SR\left(A_{n}^{i}↔A_{n^{\prime }}^{j}\right)=\;\frac{sr_{ij}}{\sqrt{V_{ij}}}$$

Here, $v_{ij}$ is the maximum likelihood estimate of the variance of $SR_{ij}$, and is defined as: $v_{ij}$ = ($1-\overset{I_{n}}{\underset{u=1}{\sum }}o_{uj}/M$) × ($1-\overset{I_{n^{\prime }}}{\underset{u=1}{\sum }}o_{iu}/M$). We should note that the respective statistical thresholds for significant and insignificant ARAs remain unchanged.

Although the SRs can reveal the significance of an ARA, subtle associations could still be entangled and masked. Hence, by treating the ARA *SR* matrix as a *SR* vector space (SRV), we have developed a novel method to disentangle the SRV through Principal Component Decomposition (PCD) onto a set of PCs and reproject the projections of the AR-vectors on each PC back to a new SRV referred to as its corresponding RSRV.

Step 2: Conduct PCD on SRV and Obtain for Each PC Its Corresponding RSRV. 

The button “PC” in Figure 3 is used to initiate the application of the PCD (Definition 3) on the SRV and obtain a number of top PCs according to the number (or the threshold of the total variance) set in the window. In the meantime, in order to disentangle the discovered AVAs from SRV, the a-vector projections on each PC is re-projected onto a new SRV, referred to as the Re-projected SRV (Definition 4). The new transformed a-vector positions in the RSRV correspond to a new set of ARA SRs for each AR with other ARs in the RSRV. These new positions of a-vectors reflect the ARAs captured in the corresponding PC.

**Definition** **3.** Principal Components. In PCD, PCs are a set of k PCs, denoted as PC = {$PC_{1},PC_{2},\ldots PC_{k}\}$, where $PC_{k}$ is a set of projections of the a-vectors from SRV, denoted as $PC_{k}$ = {$PC_{k}\left(A_{n}^{i}\right)|n=1,2,\ldots N,\;i=1,\ldots I_{n}$}, where N is the total number of all aligned columns in APC and $I_{n}$ is the total number of distinct amino acids on the column $A_{n}$.

**Definition** **4.** *Re-projected SRV (RSRV). RSRV is the SRV containing the a-vector projections on a PC. The coordinates of an a-vector projection on the PC in the RSRV represent the ARA SRs of the AR of that a-vector associating with other ARs corresponding to the column vectors as captured by the PC (Equation (3)).*

(3) $${\mathrm{RSRV}}_{k}=\mathrm{SRV}\cdot {\mathrm{PC}}_{k}\cdot {\mathrm{PC}}_{k}{}^{T}$$

After PCD, we first identify the distinct projection(s) of the a-vectors and their furthest clusters from the mean (center) of the PC-Axis. The a-vector projections in PCs with large eigenvalue should have strong association or strong presence captured by the orthogonal PCs.

**Figure 4:**
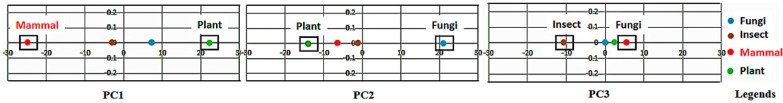
PC1, PC2 and PC3 plots for APC-6382 used as an illustrative example in Section 5. Here, only the points representing the class labels are displayed. A full plot with or without class labels is given in Figure 6.

If the class labels are included in the APCs, its position in the PCs appears just as a virtual AR (essentially the centroid of the ARs within an AR cluster pertaining to that class). In the illustrative example in Figure 4, the colored dots enclosed in the square boxes are centroids of the AR clusters associated to specific classes like mammal, plant, etc.

Hence, the new positions of the projections of the a-vectors on the PC, when transformed to the SRV, represent the a-vectors with a new set of coordinates in the RSRV. We mark the correspondence by attaching to them the same subscript, i.e., k in *PC_k_* and *RSRV_k._*

**Figure 5:**
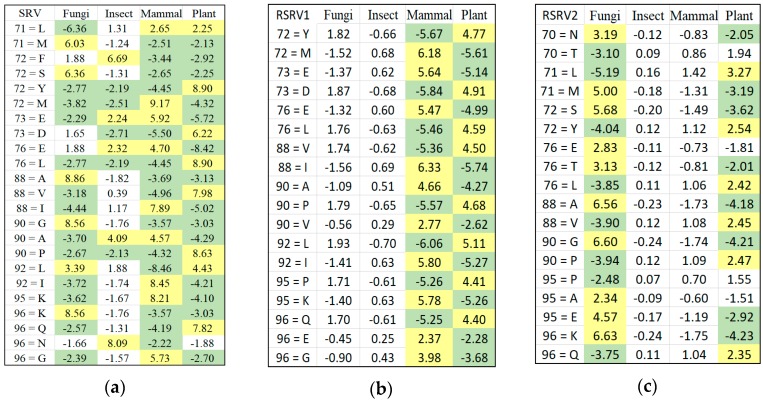
Part of SRV and RSRVs for APC-6382. (**a**) SRV shows that there is no clear partition between classes; (**b**) In RSRV1, plant and mammal are succinctly separated; (**c**) In RSRV2, plant and fungi are also distinct. Such disentanglements are captured in PC1 and PC2 as shown in Figure 4 and Figure 6.

Figure 5 shows how the SRV with AVAs, related to the taxonomical subgroups and obtained from APC-6382 (described later in the Results Section), is disentangled into different RSRVs (such as, RSRV1 and RSRV2) and thus revealing ARA subgroups corresponding to different functionality pertaining to the taxonomical classes in the region. Note that the ARAs entangled in SRV (Figure 5a) are disentangled into succinct sub-groups corresponding to difference taxonomical classes in RSRV1 and RSRV2 (Figure 5b,c, respectively).

To summarize this step, PCD uses an orthogonal transformation to transform a set of possible correlated variables into a set of linear uncorrelated variables known as PCs. The first PC, ${\mathrm{PC}}_{1}$, has the largest possible variance, which accounts for ARs with the highest ARAs with other ARs. PCs with less variance follow.

Step 3: Identify Distinct ARs and AR-Clusters Through Their Statistical Significant ARA SRs Between ARs.

**Figure 6:**
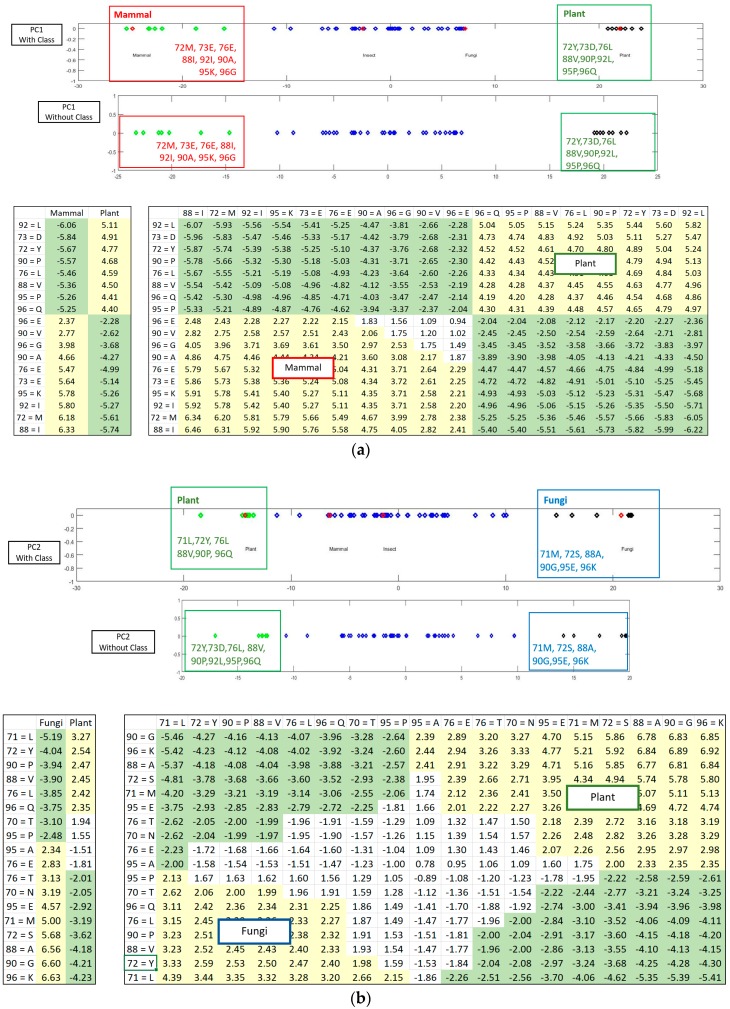
AR Clusters captured in PCs and their corresponding ARAs reflected in RSRVs for APC-6382. (**a**) RSRV1 ARA Clustering Result with and without Class Labels; (**b**) RSRV2 ARA Clustering Result with and without Class Labels. In both (**a**,**b**) cases, we observed that the AR clusters on the right-handed side RSRV plots obtained from SRV without class labels correspond closely to the class association respectively as indicated on the left-handed side plots taken from the RSRVs obtained from SRV when class labels are included. Hence, this shows that ARADD is able to obtain closely corresponding ARA patterns with or without class labels.

**Figure 7:**
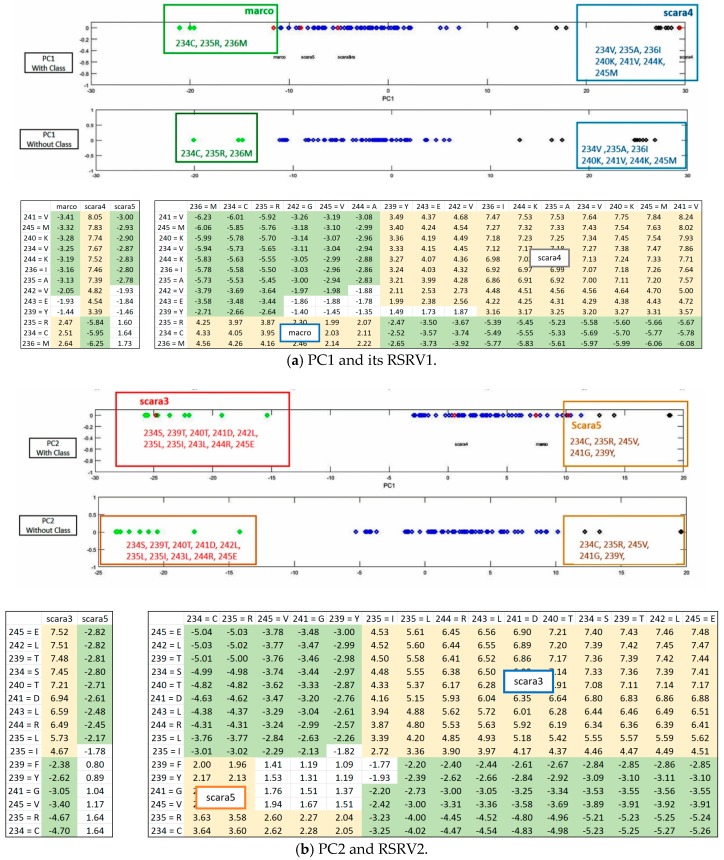
PC and RSRV results for AP2859 obtained from protein sequences of SR-A family. (**a**) PC1 and its RSRV1; (**b**) PC2 and RSRV2. Note the consistency between the AR clusters in the PCs and the a-vector groups in the RSRVs. Note also in both PC pairs, distinct AR groups associating with SR-A classes are succinctly discovered with/without the inclusion of the class labels in the APCs.

The button “Highlight Result” in Figure 3 is implemented for highlighting the results from the sub-clusters. When a cluster of ARs forms an association pattern, they should share strong ARAs. By setting the threshold to 1.96, the strong ARAs can be grouped together to represent an AR sub-cluster in the RSRVs. A careful comparison of the PCs (Figure 4) and their corresponding RSRVs (Figure 5) shows that the distinct AR clusters captured in PCs are reflected by the statistical significant ARAs in their corresponding rows (a-vectors) in the RSRVs (yellow cells). More succinct representations are found in Figure 6 and Figure 7.

Output: RSRVs and ARA Sub-Clusters

**Figure 8:**
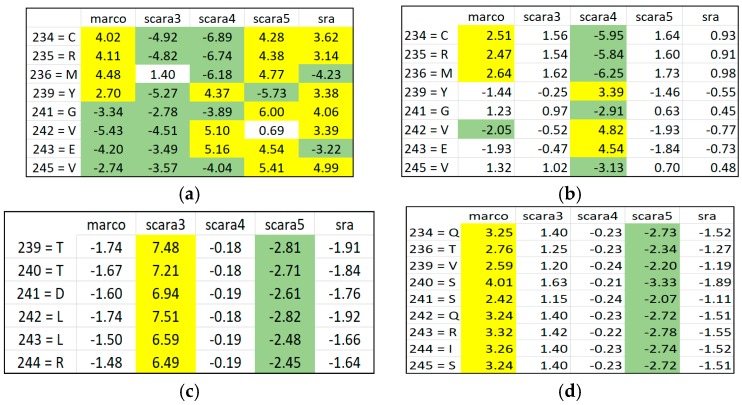
Discovered ARAs based on SRV and RSRVs by ARADD for APC-2859. (**a**) SRV where ARAs associating with class entangled; (**b**) RSRV1 with ARAs associating with Marco and Scar4 disentangled; (**c**) RSRV2 with ARAs associating with Scar3; (**d**) RSRV3 with ARAs associating with Scara5.

1. *Disentangled ARAs by distributing and grouping them in different RSRVs.* After PCD, it may not be obvious why an a-vector is significant. However, when the a-vectors are examined in the RSRVs, it can be observed that their high SR coordinate(s) contributed to their high variance on their corresponding PCs. In general, PCD is sensitive to the relative scaling of the original aligned columns. By unifying the scaling of ARA measures through SR, our results showed that SRV is rather stable and can reveal statistically significant associations between ARs with statistical strength reasonably well. However, when ARAs become entangled, the SRV disentanglement is crucial for yielding highly distinct, stable, and specific results as manifested in the RSRVs obtained from both datasets (Figure 4, Figure 5, Figure 6, Figure 7 and Figure 8).
2. *ARs Sub-clusters.* Although the disentangled PCs can already reveal significant ARs/AR-Clusters on a one-dimensional space (Figure 4, Figure 6 and Figure 8), the statistical strength (SR) of the ARAs can further reveal the significance of the ARAs and the AR-Clusters by identifying their RSRVs through the SRs from the row and column a-vectors (RSRV1 and RSRV2 on bottom half of Figure 6a,b and Figure 7a,b. By disentanglement, we can obtain different ARs sub-clusters in different orthogonal PC spaces as shown in both figures. These AR subgroups may have functional meaning. They may help to build classification or subgroup partitioning models with less features but can achieve comparably superior performance to the classification models based on statistical significant ARAs derived from SRV. Furthermore, it is much easier to select discriminative features from RSRVs after disentanglement than from SRV and from the distinct ARs and AR clusters in the PCs.

## 4. Materials

Dataset 1-APC-6382 is a cluster of aligned patterns (width: 17) covering in total 85 protein sequences consisting of 4 subclasses (Fungi, Insect, Mammal and Plant). It was one of the APCs with highest coverage obtained by applying Aligned Pattern Clustering (APCn) algorithm [16,17] on a set of 93 protein sequences of Cytochrome c obtained from [16] after preprocessing on more than 300 protein sequences. For simplicity, we selected the protein sequences belonging to Fungi, Insect, Mammal, Mammal-Primate, Mammal-Rodent, Plant, Chlorophyta and Cryptophyta. We then regrouped Mammal, Mammal-Primate, Mammal-Rodent as the same subclass Mammal, and, Plant, Chlorophyta, Cryptophyta as the same subclass Plant. This dataset is mainly for illustrating our methodology since we found that while its taxonomical classes are succinct, their aligned patterns are still somehow entangled due to their entwining common and different evolutionary functions.

Dataset 2-APC-2859 is a cluster of aligned patterns (width: 12) obtained from APCn [16,17] covering in total 95 protein sequences coming from 5 subclasses (Marco, Sra, Scara3, Scara4, Scara5) of class A scavenger receptors originally taken from a dataset with 106 sequences used in [21]. Among all APCs, APC-2859 is the one with the highest coverage. All five subclasses of proteins contain domains: Cytoplasmic, Collagenous, Transmembrane, a-helical and coiled-coil motifs. Marco, Sra, and Scara5 contain the Collagenous domain. Only Sra contains the SRCR domain.

Here we first report our experimental results on Dataset 1-APC-6382 from Cytochrome c as an illustrative example of the proposed methodology. We then report our experimental results on Dataset 2-APC-2859 from class A scavenger receptors to reveal ARADD capability of discovering, disentangling and locating much more subtle subgroup characteristics entangled in the source environment.

## 5. Experimental Results

### 5.1. Experimental Results on Dataset 1-APC-6382 of Sequences from Cytochrome C Family

We used APC-6382 obtained from the Cytochrome c protein family with taxonomic class labels to illustrate how ARADD works. We first constructed SRVs between each pair of ARs in the APC dataset. The $SR(A_{n}^{i}↔A_{n}^{i}$) with zero value corresponds to the co-occurrence of the same AR and therefore has no meaning. The value of SRs which is above or below ±1.96 represent respectively positive or negative significant ARAs and are shaded respectively in yellow and in green. Figure 5a shows the SRV result for the association of ARs with class labels if they are included in the data space of the APC. We found that in many cases, the entangled ARs cannot distinguish different classes. For example, as a case of ARA entanglement from different classes, “AR71 = L” is associated with both Mammal and Plant but is highlighted as the disentangled result given in the SRV in Figure 5.

We then applied PCD on the SRV obtained from APCs with class labels attached and obtained PCs ranked after their variance. We projected the a-vector projections on the PCs to RSRVs to obtain a new set of coordinates that are the SRs of each ARA between ARs corresponding to the row and column a-vectors. Figure 5 shows the disentanglement of the class labels. Figure 6a,b show the ARs made up of distinct clusters in the PCs and their corresponding ARAs from their a-vectors in the RSRVs. We conclude that the entangled ARAs in SRV are disentangled in the RSRVs.

From the above experimental result, we conclude that when an APC with class labels is given as input, we could detect different groups of ARs related with class labels on different PCs and their ARAs on their corresponding RSRVs. The RSRVs results could succinctly reveal ARAs sharing with or discriminating from different subgroups conditioned (or governed) by certain biological functions on that spot. In order to show that such functional associations are intrinsic even if the class labels are not included, we conduct separate experiments on APCs with and without class labels included.

On each PC pair (with class labels or without) on top of Figure 6a,b respectively, we observed that ARs that made up of the AR clusters corresponding to taxonomical classes remain essentially the same, though their positions have changed a little due to the change in statistics, resulting from the presence or absence of the class labels. The results in both Cytochrome c (Figure 6) and SR-A (Figure 7) support such claim. Note that on the plot with class label included, the red diamonds represent the class label when treated as an attribute with its values representing different classes. This shows that ARADD is robust in discovering and disentangling ARAs with or without the explicit reliance on class labels. Hence, it is effective for supervised classification and unsupervised subgroup analysis without explicit reliance on prior knowledge, a significant advantage in mining large volume of omitted data.

### 5.2. Experimental Results on Dataset 2-APC-2859 from Class A Scavenger Receptor Family Sequences

To meet the challenge of a well-recognized difficult proteomic problem, we applied ARADD on APC-2859 obtained from sequences of SR-A, the dataset 2 as described in Section 4. First, we compared the discovered ARAs obtained in RSRVs with those using only the adjusted statistical residual (SR) in SRV [1] with the same threshold SR > 1.96 all through (Figure 8).

Figure 8a shows the discovered ARAs only using SRV result for APC-2859. In this dataset, ARAs in different classes discovered are all included in the APC due to their similarity. However, we observed that their ARAs and class relationship are entangled, implying that the patterns in the APC are also entangled. When we checked the discovered ARAs obtained through using ARADD in different RSRVs, we observed the disentangled results as shown succinctly in Figure 8b–d.

In Figure 8b, if we select those entangled residue values in SRV from RSRV1, we found that in SRV, Marco is entangled with Scara5 and Sra, while in RSRV1, Marco is disentangled from Scara5 and Sra among residues in aligned sites 234, 235 and 236. In Figure 8c,d, Scara5 and Marco are disentangled and manifested as distinct groups from other classes.

Table 1 shows the AR subgroups discovered in the APC sequence pattern space plotted on the APC data space. The ARs with statistical significant ARAs with other ARs are in bold colored fonts. The first and the last column tabulate the sequence IDs and sequence positions range of the a-vectors respectively. Note that the AR pattern for Scara5 is CRM****G***V and that for Sra is CR***Y*G***V, which are similar but mapped onto two distant domains. Hence, ARADD not only can disentangle functional association in the pattern space but also disentangle their sequence location that is related to different domains of the family.

From the experimental results, we found that ARADD not only can discover the statistically significant ARAs, though entangled in the SRV, but much more novel and crucial is that it can discover significant ARs and/or AR Clusters (ARCs) captured in orthogonal PCs to bring out their separability as shown in Figure 8a,b respectively. We note that class Scara4 stands out in RSRV1; and Scara3 and Scara5 are two distinct subgroups, one as an opposite in RSRV2; and Scara3 and Scara4 are separated in RSRV3.

To show that the disentangled results remain intact with minor changes in AR position on APC when class labels are absent, we apply ARADD to the same set of data with class labels removed.

Figure 8 also shows from the PCs the closeness of the ARs found in each succinct cluster with and without class label in respective PC1 and PC2 (Figure 8a,b). We observed that that the AR cluster configurations have little change in their respective PC spaces. When class labels are included they appear in the PCs as a projection denoted by the red diamonds.

## 6. Discussion

**Figure 9:**
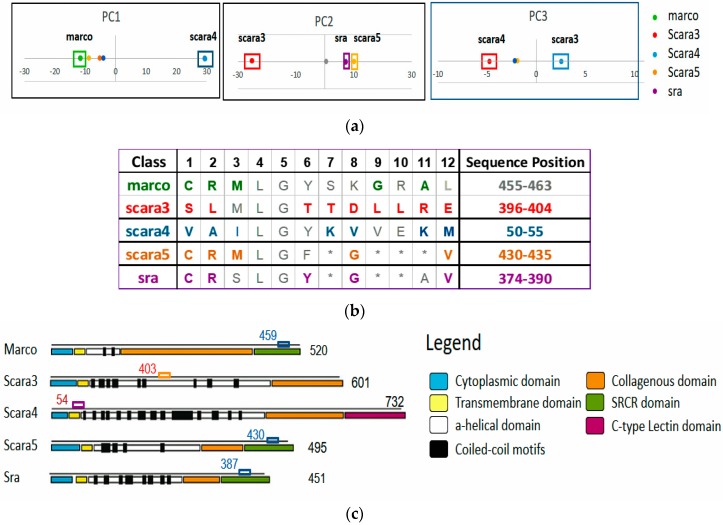
ARA disentanglement in pattern space and sequence location. (**a**) Disentanglement of functional groups corresponding to class displayed by their class labels; (**b**) Typical AR Groups in distinct bold colored ARs corresponding to different classes with sequence ID and the range of positions listed; (**c**) A mapping of the 5 patterns (AR groups) onto the protein sequences with domain regions annotated described in the legends [1] and class labels associating with the patterns. It shows the sequence mean position of the patterns (in small colored boxes and sequence position indices) and the sequence lengths in black digits at the end the sequences. Though patterns were clustered in the same APC due to sequence similarity, the ARA patterns reside in different domain corresponding to different biological function. Patterns found by ARADD fall into the range of each domain.

The tabulated results in Table 1 give strong scientific support to the significance of ARA disentanglement in proteomic research, revealing the crucial information of “what” and “where” in a protein family. Figure 9 gives a succinct view of the discovered results both in pattern and data space. Figure 9a shows that the class labels associating with ARs of that class are discovered within their associating clusters in the one-dimensional disentangled PC space. As revealed in Table 1, the AR groups for Scara5 and Sra are very close with only a single difference in their significant ARs. Their closeness is also revealed in RSRV2. Both deviate significantly from Scara3. Hence, from the PCs (Figure 9a) and the plots of the significant AR clusters (pattern space) we have at a glance of their similarity and differences with statistical backing. From the APC data space in Table 1, we observed that both the sequence ID and sequence position of each of the AR pattern were revealed. They are surprisingly closely correlated with the domain regions annotated the legends of a figure taken from [1]. Table 2 summarizes the sequence class and position information of the discovered patterns we obtained through ARADD and details how they correspond to domain regions obtained from biological experiment as reported in [1].

The results for SR-A are more profound than what we perceive on the surface. The experimental result we present in this paper provides significant evidence to support our conjecture that the discovered subtle deep knowledge entangled in the source environment and masked on the surface of the observed data can be discovered by ARADD without explicit reliance on prior knowledge. In a nut shell, ARADD [4] is able to reveal and locate the significant AR clusters, the “what” and “where” of subtle functional groups. The former is revealed through the disentangled PCs and RSRVs while the latter through the address table assembled during the significant ARA discovery process. Hence, we first introduce the notion on the ARA pattern space and the data space of an APC [16,17] before we dive into the experiments and the results. The former expresses which ARs, AR clusters, ARAs and ARA clusters are functionally and statistically significance through their association strength in the PCs and RSRVs. The latter displays where they are in the family sequences and relating them to the family domains found or validated through biology experiments. Hence, two functional groups with similar ARAs (like Scara5 and Sra, with the only difference in sites 236 and 239 discovered by ARADD represent different functional groups occurring in different regions (sequence position) of the family (shown in last column of Table 1 and Figure 9c).

### Expanded Discussion of Protein Sequence Analysis: A Comparison with MEME

To investigate if the sequence patterns discovered and clustered by other models have been trapped in the entanglement of biological functions of similar ARAs due to unknown factors or carrying different functionality in different domain locations, we conducted an experiment referred to as Experiment 6A. We used a popular motif discovery algorithm, MEME [7], to obtain motifs on Dataset 2 as a case study. Later in Experiment 6B, we showed that the exhibition of AR clusters for different subclasses, with their positions located in the family sequence domains, can be obtained via disentanglement through ARADD.

In Experiment 6A, we first applied MEME [7] on Dataset 2, covering in total 95 protein sequences coming from five subclasses (Marco, Sra, Scara3, Scara4, Scara5) of class A scavenger receptors. The details for Dataset 2 could be referred to Section 4 on Materials. The parameter setting was as follows: minimum width = 10, maximum width = 15, number of motifs = 30. These parameters were set to find motifs with comparison to APC-2859.

**Figure 10:**
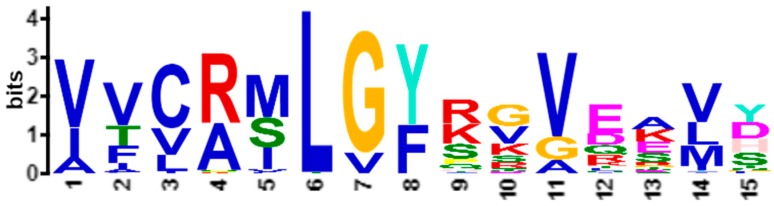
The fifth-ranked motif obtained by running MEME on Dataset 2. The motif covers all 95 sequences, with an E-value of 1.7 × 10^−690^.

After running MEME on Dataset 2, we obtained 30 motifs in the form of PWM’s [6]. We found that the fifth-ranked motif was most similar to APC-2859. This motif covering all 95 sequences, with an E-value of 1.7 × 10^−690^, is depicted in Figure 10. All sequence patterns that compose the motif (Figure 10) are summarized in the Supplementary Table S1.

By averaging the starting position of the sequence patterns (Supplementary Table S1) that compose the motif depicted in Figure 10, we report that the domains where the average starting positions of these patterns reside correspond to the five subclasses (Marco, Sra, Scara3, Scara4, Scara5) of class A scavenger receptors domains as shown in Table 3.

As shown in Table 3, we observed that the sequence patterns discovered and clustered by MEME [7] clearly show the entanglement phenomena of biological functions as similar ARAs are occurring in different domain location. In other words, although the sequence patterns are discovered and clustered in the same motif by MEME [7], they actually reside in different biological domains and thus may have a little different biological functions.

**Figure 11:**
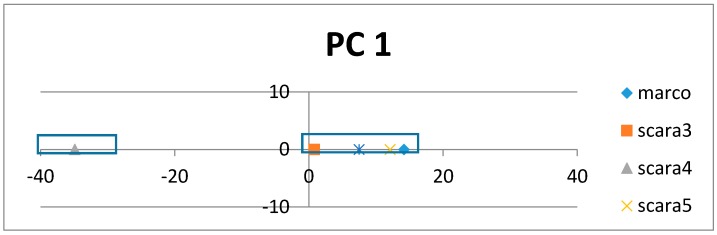
The 1st PC projection plot obtained by applying ARADD algorithm on the APCs of sequence patterns as summarized in Supplementary Table S1. It should be noted that the pattern occurrence position (indicated by the column “start” in Table S1) was not inputted to ARADD algorithm.

**Figure 12:**
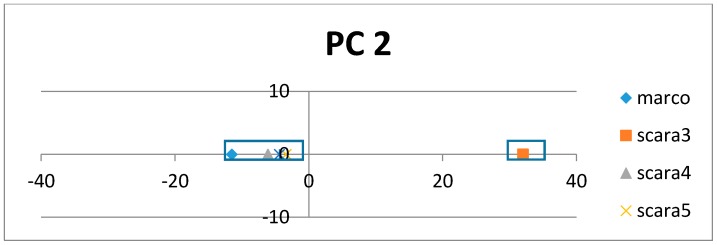
The 2nd PC projection plot obtained by applying ARADD algorithm on the APCs of sequence patterns as summarized Supplementary Table S1. It should be noted that the pattern occurrence position (indicated by the column “start” in Table S1) was not inputted to ARADD algorithm.

In Experiment 6B, we applied ARADD to the APCs of sequence patterns as summarized in Supplementary Table S1 and obtained the PC projection plots as depicted in Figure 11 and Figure 12. We found that, in the PC projection plots, the domain information corresponding to the subclasses was reflected exactly as reported in [1], while neither the pattern occurrence position nor the domain information were inputted into the ARADD algorithm. We observed that the subclasses Scara4 and Scara3 are distinctly revealed in PC1 (Figure 11) and PC2 (Figure 12) respectively. This is consistent to the biological domain distinction shown in Table 3. In other words, ARADD algorithm has successfully disentangled the sequence patterns composing the motif (Figure 10) not relying on the knowledge of the pattern occurrence positions.

The above results pointed out that not only in APCs but also in motifs obtained by MEME [7], the sequence patterns that were discovered and clustered could be entangled in biological functions and domain location. These results show that in both scenarios [4,7], ARADD is able to disentangle the sequence patterns to reflect the domain information corresponding to different subclasses without knowing the actual pattern occurrence positions.

## 7. Conclusions

By applying the ARADD algorithm [4] to an entangled APC obtained from class A scavenger receptor, this study has shown that AR clusters (patterns), associating with different functional subgroups, regions and domains of the family obtained from an APC, could be succinctly plotted and statistically separated in different PCs and RSRVs as well as in different location through their sequence ID and sequence position of the family.

The most significant finding of this study is that the aligned patterns could be disentangled into ARA subgroups associating with different classes or subgroups, residing in different functional regions or domains of the family, even within an APC where conserved functional segments from different sequences are aligned. Such findings are absent in existing sequence alignment methods, but clearly revealed in our study on the class A scavenger receptor family.

Biologically, entangled patterns in class A scavenger receptor that are aligned in a sequence conservation model, such as in an APC, reveal biological functional patterns pertaining to similar or different classes. Entangled patterns can be disentangled into subgroups pertaining to different functionality, such as class A scavenger receptor classes into ARA subgroups mapped onto different PCs and RSRVs and located in different functional domains of the class A scavenger receptor family. Therefore, the successful application of ARADD algorithm to class A scavenger receptor demonstrates its capability to open a new way for analyzing conserved regions and their distribution, with potential to reveal new knowledge for gene therapy applications [2].

## Figures and Tables

**Table 1:**
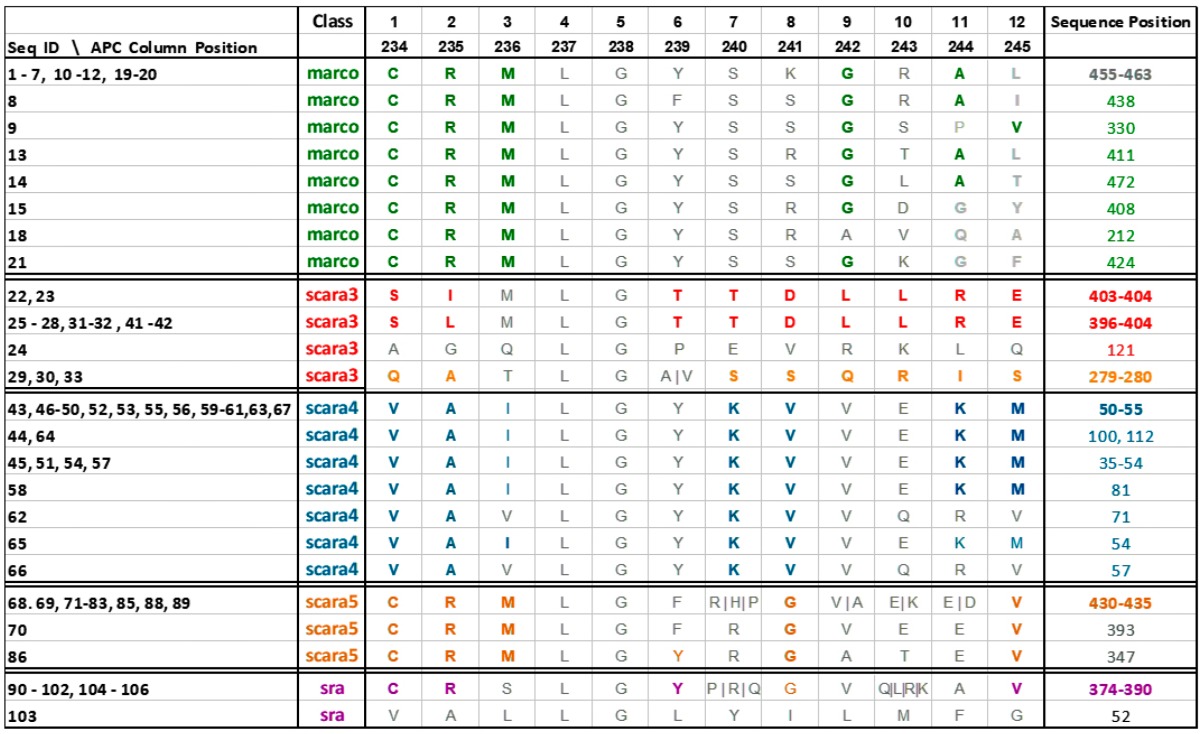
Experimental results of AR groups associating with different SR-A classes. The first column tabulates the sequence IDs of the AR groups in the data space. The second column tabulates the SR-A Class with which each AR group is associating. The AR in bold on the third column indicates that it is the ARs in an a-vector with strong SR with other ARs. The last column tabulates the range of the sequence position on which each AR groups resides.

**Table 2:** A summary of the occurrences of the discovered patterns and their position in different class sequences and their correspondence with the biology domains reported in [1].

Covering Sequences Subclasses	Average Pattern Occurrence (or Address) Position	Domain
Marco	431.5	SRCR
Sra	367.8	SRCR
Scara5	423.7	SRCR
Scara4	58.0	Transmembrane/alpha-helical with coil-coiled motifs
Scara3	355.9	alpha-helical with coil-coiled motifs

**Table 3:** A summary of the occurrences of the discovered patterns by MEME [7] and their position in different class sequences and their correspondence with the biology domains reported in [1]. The problem here is that these sequence patterns are clustered in the same motif, but they actually occur in different biological domains.

Covering Sequences Subclasses	Average Pattern Occurrence (or Address) Position	Domain
Marco	425.1	SRCR
Sra	367.6	SRCR
Scara5	422.7	SRCR
Scara4	55.6	Transmembrane/alpha-helical with coil-coiled motifs
Scara3	69.6	alpha-helical with coil-coiled motifs

## Supplementary Materials

The following are available online http://www.mdpi.com/2227-7382/6/1/10/s1, Supplementary document 1: The software executable used in this study and a set of test data; Supplementary Table S1: All sequence patterns that compose the motif (Figure 10).

## References

[1] Whelan F.J., Meehan C.J., Golding G.B., McConkey B.J., Bowdish D.M.E. (2012). The evolution of the class A scavenger receptors. BMC Evol. Biol..

[2] Zani I.A., Stephen S.L., Mughal N.A., Russell D., Homer-Vanniasinkam S., Wheatcroft S.B., Ponnambalam S. (2015). Scavenger receptor structure and function in health and disease. Cells.

[3] Plüddemann A., Mukhopadhyay S., Sankala M., Savino S., Pizza M., Rappuoli R., Tryggvason K., Gordon S. (2009). SR-A, MARCO and TLRs differentially recognise selected surface proteins from neisseria meningitidis: An example of fine specificity in microbial ligand recognition by innate immune receptors. J. Innate Immun..

[4] Zhou P., Wong A.K.C., Sze-To A. Discovery and Disentanglement of Protein Aligned Pattern Clusters to Reveal Subtle Functional Subgroups. Proceedings of the 2017 IEEE International Conference on Bioinformatics and Biomedicine (IEEE BIBM 2017).

[5] Wong A.K.C., Zhou P., Sze-To A. Discovering Deep Knowledge from Relational Data by Attribute-Value Association. Proceedings of the 13th International Conference on Data Mining (DMIN’17).

[6] Xia X. (2012). Position Weight Matrix, Gibbs Sampler, and the Associated Significance Tests in Motif Characterization and Prediction. Scientifica (Cairo).

[7] Bailey T.L., Boden M., Buske F.A., Frith M., Grant C.E., Clementi L., Ren J., Li W.W., Noble W.S. (2009). MEME Suite: Tools for motif discovery and searching. Nucleic Acids Res..

[8] Edgar R.C., Batzoglou S. (2006). Multiple sequence alignment. Curr. Opin. Struct. Biol..

[9] Thompson J.D., Linard B., Lecompte O., Poch O. (2011). A comprehensive benchmark study of multiple sequence alignment methods: Current challenges and future perspectives. PLoS ONE.

[10] D’haeseleer P. (2006). How does DNA sequence motif discovery work?. Nat. Biotechnol..

[11] Altschuh D., Lesk A.M., Bloomer A.C., Klug A. (1987). Correlation of co-ordinated amino acid substitutions with function in viruses related to tobacco mosaic virus. J. Mol. Biol..

[12] Kass I., Horovitz A. (2002). Mapping pathways of allosteric communication in GroEL by analysis of correlated mutations. Proteins Struct. Funct. Genet..

[13] Chau T., Wong A.K.C. (1999). Pattern discovery by residual analysis and recursive partitioning. IEEE Trans. Knowl. Data Eng..

[14] Wang Y., Wong A.K.C. (2003). From association to classification: Inference using weight of evidence. IEEE Trans. Knowl. Data Eng..

[15] Jiawei H., Kamber M., Han J., Kamber M., Pei J. (2012). Data Mining: Concepts and Techniques.

[16] Lee E.-S., Wong A.K. (2013). Ranking and compacting binding segments of protein families using aligned pattern clusters. Proteome Sci..

[17] Wong A.K.C., Lee E.S.A. (2014). Aligning and clustering patterns to reveal the protein functionality of sequences. IEEE/ACM Trans. Comput. Biol. Bioinform..

[18] Naulaerts S., Meysman P., Bittremieux W., Vu T.N., Vanden Berghe W., Goethals B., Laukens K. (2015). A primer to frequent itemset mining for bioinformatics. Brief. Bioinform..

[19] Agrawal R., Imielinski T., Swami A. Mining Association in Large Databases. Proceedings of the 1993 ACM SIGMOD International Conference on Management of Data.

[20] Han J., Pei J., Yin Y., Mao R. (2004). Mining frequent patterns without candidate generation: A frequent-pattern tree approach. Data Min. Knowl. Discov..

[21] Lee E.-S.A., Whelan F.J., Bowdish D.M.E., Wong A.K.C. (2016). Partitioning and correlating subgroup characteristics from Aligned Pattern Clusters. Bioinformatics.

